# The impact of nonverbal ability on prevalence and clinical presentation of language disorder: evidence from a population study

**DOI:** 10.1111/jcpp.12573

**Published:** 2016-05-16

**Authors:** Courtenay Frazier Norbury, Debbie Gooch, Charlotte Wray, Gillian Baird, Tony Charman, Emily Simonoff, George Vamvakas, Andrew Pickles

**Affiliations:** ^1^ Psychology and Language SciencesUniversity College LondonLondonUK; ^2^ Department of PsychologyRoyal HollowayUniversity of LondonLondonUK; ^3^ Newcomen CentreSt Thomas’ HospitalLondonUK; ^4^ Department of PsychologyInstitute of Psychiatry, Psychology and NeuroscienceKing's College LondonLondonUK; ^5^ Department of Child and Adolescent PsychiatryInstitute of Psychiatry, Psychology and NeuroscienceKing's College LondonLondonUK; ^6^ Department of BiostatisticsInstitute of Psychiatry, Psychology and NeuroscienceKing's College LondonLondonUK

**Keywords:** Developmental language disorder, NVIQ discrepancy, prevalence, functional impairment

## Abstract

**Background:**

Diagnosis of ‘specific’ language impairment traditionally required nonverbal IQ to be within normal limits, often resulting in restricted access to clinical services for children with lower NVIQ. Changes to DSM‐5 criteria for language disorder removed this NVIQ requirement. This study sought to delineate the impact of varying NVIQ criteria on prevalence, clinical presentation and functional impact of language disorder in the first UK population study of language impairment at school entry.

**Methods:**

A population‐based survey design with sample weighting procedures was used to estimate population prevalence. We surveyed state‐maintained reception classrooms (*n* = 161 or 61% of eligible schools) in Surrey, England. From a total population of 12,398 children (ages 4–5 years), 7,267 (59%) were screened. A stratified subsample (*n* = 529) received comprehensive assessment of language, NVIQ, social, emotional and behavioural problems, and academic attainment.

**Results:**

The total population prevalence estimate of language disorder was 9.92% (95% CI 7.38, 13.20). The prevalence of language disorder of unknown origin was estimated to be 7.58% (95% CI 5.33, 10.66), while the prevalence of language impairment associated with intellectual disability and/or existing medical diagnosis was 2.34% (95% CI 1.40, 3.91). Children with language disorder displayed elevated symptoms of social, emotional and behavioural problems relative to peers, *F*(1, 466) = 7.88, *p *=* *.05, and 88% did not make expected academic progress. There were no differences between those with average and low‐average NVIQ scores in severity of language deficit, social, emotional and behavioural problems, or educational attainment. In contrast, children with language impairments associated with known medical diagnosis and/or intellectual disability displayed more severe deficits on multiple measures.

**Conclusions:**

At school entry, approximately two children in every class of 30 pupils will experience language disorder severe enough to hinder academic progress. Access to specialist clinical services should not depend on NVIQ.

## Introduction

Developmental language disorder is a public health concern (Law, Reilly, & Snow, 2013), associated with increased risk of school failure (Tomblin, 2008), poor employment outcomes (Conti‐Ramsden & Durkin, 2012) and social, emotional, and behaviour problems (Yew & O'Kearney, 2013). Estimating prevalence and planning services for children with language disorder is hampered by a lack of consensus concerning key inclusion and exclusion criteria (Bishop, 2014). There is little agreement regarding the level of language deficit that results in functional impairment and considerable debate about the role of NVIQ in diagnosis and treatment. For example, ICD‐10 criteria for language disorders (World Health Organisation, 1992) specify severe language deficits (−2*SD* or more) in the context of average NVIQ, yielding a significant discrepancy between verbal and nonverbal abilities. Similarly, NVIQ below the average range (below −1*SD*, equivalent to standard scores below 85 and in some cases below 90) is the most common exclusion criterion for admission to specialist speech‐language therapy services in England (Dockrell, Lindsay, Letchford, & Mackie, 2006) and Ireland (Dept for Education, Co. Westmeath, 2005), regardless of the severity of language impairment. This creates a group of children with considerable language needs who fall between diagnostic categories because they do not meet criteria for specific language intervention services (because their nonverbal abilities are too impaired), nor do they meet criteria for education provisions catering for children with learning disabilities (because their NVIQ deficits are not severe enough). The 5th revision of the Diagnostic and Statistical Manual of Mental Disorders (DSM‐5: American Psychiatric Association, 2013) removed reference to NVIQ in the criteria for developmental language disorder, providing children do not meet the criteria for intellectual disability. Such differences in diagnostic criteria may yield substantially different prevalence estimates, and potentially identify children with disparate clinical needs.

Existing language and NVIQ criteria are entirely arbitrary, as the extent to which diagnostic criteria are associated with functional impacts in education or social, emotional and behavioural development has not been systematically tested in the general population. An investigation of children at increased biological or psychosocial risk of language disorder considered varying language and NVIQ criteria on prevalence (Weindrich, Jennen‐Steinmetz, Laucht, Esser, & Schmidt, 2000). Strict application of ICD‐10 criteria yielded a prevalence estimate of 2.2% at age 4, while broadening criteria to include children with a language deficit of −1.5*SD* and low‐average NVIQ (scores between −2*SD* and −1*SD*) trebled the prevalence estimate. Importantly, children meeting ICD‐10 criteria were most likely to spontaneously resolve language deficits by age 8, while peers with low‐average NVIQ had persistent language disorders and were more likely to develop literacy problems.

Poor prognosis in this study may have been confounded by the presence of additional risk factors. Population studies are necessary to explore the relationship between language, NVIQ and functional impact in unbiased cohorts. To date, previous investigations have either excluded children with low‐average NVIQ, did not compare those with average and low‐average IQ, or failed to measure functional impact (Beitchman, Nair, Clegg, & Patel, 1986; Eadie et al., 2014; McLeod & Harrison, 2009; Silva, McGee, & Williams, 2008; Stevenson & Richman, 1976; Tomblin et al., 1997). In addition, the severity of language deficit required to meet criteria for disorder varies widely, affecting prevalence estimates. The most commonly cited prevalence estimate of 7.4% (Tomblin et al., 1997) required an overall language deficit of −1.12*SD* in the context of average (standard score > 87) NVIQ. However, these criteria identified a large number of false positives, with fewer than 50% of children meeting the same criteria for language disorder 1 year later (Tomblin, Zhang, Buckwalter, & O'Brien, 2003), and only 29% attracting parent or clinical concern (Zhang & Tomblin, 2000).

The use of NVIQ as an exclusionary criterion for language disorder has been questioned (Reilly, Bishop, & Tomblin, 2014). There are consistent relationships between severity of language disorder and lower NVIQ (Conti‐Ramsden, St Clair, Pickles, & Durkin, 2012; Gallinat & Spaulding, 2014), no aetiological differences between those with and without discrepant abilities (Bishop, North, & Donlan, 1995), and no evidence that children with low‐average NVIQ cannot benefit from clinical interventions (Reilly, Bishop, et al., 2014); such findings have motivated the DSM‐5 change in diagnostic criteria for language disorder. This change has raised concerns that higher prevalence rates will increase burden on clinical and educational services to accommodate children with more severe, persistent and pervasive language disorder (Reilly, Tomblin, et al., 2014). Evidence concerning the influence of NVIQ on quantitative or qualitative differences in the clinical presentation of children with language disorder is urgently needed. In addition, while classification systems such as DSM‐5 specify a criterion of functional impairment, none delineate how this should be operationalized, and no previous epidemiological study has implemented a measure of functional impact.

Our study is the first UK population study of language disorder at school entry and the first to attempt to implement DSM‐5 criteria. We consider for the first time the functional impact of language disorder with particular focus on academic achievement. In addition, we compare children with average and low‐average NVIQ scores to estimate the influence of NVIQ on severity of language deficit, associated social, emotional and behavioural problems, and related functional disorder during the first school years.

## Methods

### Study population

**Figure 1:**
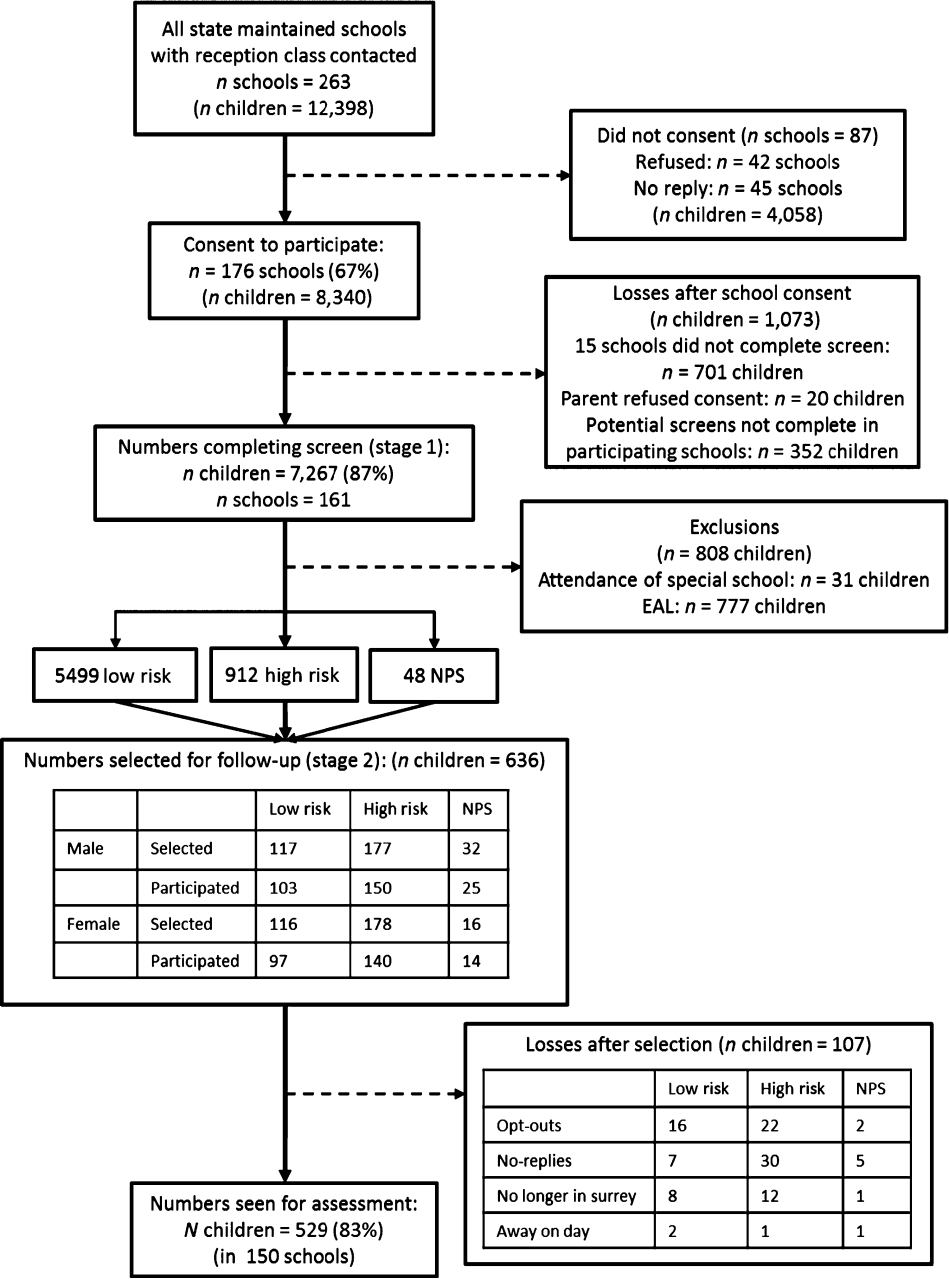
CONSORT flow diagram of recruitment and participation. High‐risk cut‐off was top 14% score for age group (autumn, spring, summer) and sex. NPS, no phrase speech; EAL, English as an additional language

The Surrey Communication and Language in Education Study (SCALES) used a two‐phase design. In the first phase, all state‐maintained primary schools in Surrey, England were invited (*n* = 263 schools) and data were obtained for 7,267 children who began a reception class (similar to kindergarten or school entry) in 2011 (response rate: 61% of all eligible schools and 59% of all eligible children, Figure 1). Participating schools (*n* = 161) did not differ from those that opted out (*n* = 102) on measures of socioeconomic disadvantage (percentage of children receiving free school meals, *t*(261) = 1.38, *p* = .17); children in receipt of a statement of special educational need (a legal document stipulating the services the local education authority is required to provide to support language, learning or behavioural problems within school), *t*(261) = 0.19, *p* = .85; or children speaking English as an additional language, *t*(232) = 1.05, *p* = .29. All children were aged between 4 years; 9 months and 5 years; 10 months at the time of assessment, in the summer (3rd) term. Although Surrey is a relatively affluent county compared with the national average, children were screened from across the social strata. Income Deprivation Affecting Children Index scores obtained from home postcodes provided a measure of socioeconomic status reflecting neighbourhood deprivation (McLennan, Barnes, Davies, Garratt, & Dibben, 2011). Index scores in England range from 1 (most deprived) to 32,844 (mean for England in 2010 = 16,241), and in this sample ranged from 731 (most deprived) to 32,474 (most affluent) (mean = 21,592, *SD* = 7,830).

In the second phase, a subsample was selected for in‐depth assessment in Year 1 (first grade, ages 5;1 – 6;10) using stratified random sampling. Initial strata identified children who were reported as having ‘no phrase speech’ (NPS, *n* = 89, 1.2%), those attending special schools for severe and complex learning disabilities (*n* = 31, including 19 NPS, 0.4%) and those for whom English was an additional language (*n* = 782, 10.7%, including 27 NPS). Children in special schools were excluded from further study; given their complex learning disabilities school staff felt they would be unable to participate in the assessments. Children with English as an additional language were invited to a different study and not included here. All remaining children with NPS (*n* = 48) were invited for in‐depth assessment.

For remaining monolingual children, cut‐off scores on the teacher‐rated Children's Communication Checklist‐Short (CCC‐S; from CCC‐2, Bishop, 2003a) were used for each of three age‐groups (autumn, spring and summer born) to identify sex‐specific strata of boys (13.9%) and girls (14.8%) with likely poorer language for age and sex (roughly equivalent to 1*SD* above expected range for sex and age group). At this stage we used sex‐specific cut‐off scores, in part because of the significant increase in summer‐born boys rated by teachers as having potential language deficits. In addition to the 48 children with NPS, a random sample of 588 were drawn from the total 6411 with a higher sampling fraction for the high‐risk children (40.5% boys, 37.5% girls) versus low‐risk children (4.3% boys, 4.2% girls). Although boys were identified in Phase 1 as high‐risk at a ratio of 2:1 (Norbury et al., 2016), we oversampled girls to ensure sufficient numbers of both sex to address potential sex differences in prevalence rates and/or clinical profile. However, our weighting procedures take account of this sampling design (i.e. boy scores carry greater ‘weight’) and therefore reported estimates reflect the entire screened sample distribution.

### Consent procedures

Opt‐out consent was adopted for the first phase as data could be provided anonymously to the research team; 20 families opted out. In the second phase of direct assessment, written, informed consent was obtained from the parents or legal guardians of all participants. Consent procedures and study protocol were developed in consultation with Surrey County Council and approved by the Research Ethics Committee at Royal Holloway, University of London.

### Screening procedures

Screening data were obtained between May–July 2012. The primary screen was the teacher‐completed CCC‐S, a brief version of the CCC‐2 (Bishop, 2003a), which contains 13 items rated on a 4‐point scale that best discriminated cases and controls in a validation study (Norbury, Nash, Baird, & Bishop, 2004). Scores ranged from 0 to 39; higher scores reflect greater disorder. Children with ‘no phrase speech’ (NPS), that is, not yet combining words into phrases or sentences, received the maximum score of 39 (1.2% of total population).

Teachers also completed the Strengths and Difficulties Questionnaire (SDQ: Goodman, 1997), a well‐validated questionnaire rating social, emotional and behavioural (SEB) strengths and weakness. The Total Difficulties subscale contains 20 items rated on a 3‐point scale, with higher scores reflecting increased difficulties (maximum score = 40), and a cut‐off score of 16 or greater indicative of clinically significant SEB problems (most extreme 10% of validation sample, Goodman, 1997). Functional impact was measured using the Early Years Foundation Stage Profile (EYFSP: Department for Education, 2013), a nationally applied measure of academic attainment for children attending state‐maintained reception classes in England. Children achieving a ‘good level of development’ made expected or exceeded progress on 12 key curriculum targets (Cotzias & Whitehorn, 2013). Teachers also reported existing clinical diagnoses (e.g. autism spectrum disorder, Down syndrome) and receipt of extra educational support, including school action (teachers provide additional input and measure progress), involvement of external agencies (referral to speech‐language therapy or educational psychology) and/or a statement of special educational need (a legally binding document specifying educational support required).

### In‐depth assessment procedure

Assessment data were obtained from 39/48 children with NPS (87.5% of girls, 78.0% of boys) and 490/588 children with phrase speech (80.3% of girls and 80.6% of boys) whose families gave consent for participation. This represents 3.6% low‐risk girls, 3.7% low‐risk boys, 31.4% high‐risk girls and 31.5% high‐risk boys from the total population screened.

In‐depth assessment closely followed procedures which have informed DSM‐5 diagnostic criteria (see supplementary material for descriptions of all assessment measures). NVIQ was estimated using a composite of block design and matrix reasoning (Wechsler, 2003). Children were banded according to IQ performance as ‘average’ (−1*SD* or better), ‘low‐average’ (between −1*SD* and −2*SD*) and ‘intellectual disability’ (−2*SD* or lower), reflecting previous research criteria and current educational practice. Speech intelligibility was assessed using a single word speech sample and reported as per cent consonants correct (Dodd, Zhu, Crosbie, Holm, & Ozanne, 2002). Five language composites included expressive and receptive vocabulary (Brownell, 2010); receptive and expressive grammar (Bishop, 2003b, Marinis, Chiat, Armon‐Lotem, Piper, & Roy, 2011); narrative retelling and comprehension (Adams, Cooke, Crutchley, Hesketh, & Reeves, 2001); and expressive and receptive composites comprised of the relevant vocabulary, grammar and narrative indices (see supporting information). Language disorder was defined as scores of −1.5*SD* or below on two of five language composites in the absence of intellectual disability and/or existing medical diagnosis. This cut‐off is consistent with earlier population studies of language disorder (Silva et al., 2008; Stevenson & Richman, 1976), studies of language disorder in other clinical populations (Loucas et al., 2008) and current clinical practice.

### Statistical analysis

Statistical analyses were conducted using *svy* procedures in Stata‐14 (Stata Corporation, 2015). Since the probability of being included in the second stage depended on language‐level (we oversampled those with language deficits), sex (we oversampled girls), and school size (larger schools were more likely to be selected), the data from the second stage participants were weighted by the inverse of the probability of selection. This weighted sample thus ‘looked like’ the first stage sample giving proportions, percentages and means that were estimates for the whole population of monolingual children starting school in state‐maintained schools. Confidence intervals and test statistics were based on robust standard errors that properly reflected sampling variability in weighted estimates. Since several measures had no, out‐of‐date or inapplicable published norms, the raw scores on these assessments were adjusted for child age and were standardised using the current weighted sample using the LMS procedure (Cole & Green, 1992) (similar to the procedures used to construct paediatric height and weight charts). These are reported as *z*‐scores with a mean of 0 and a standard deviation of 1.

### Missing data

Household postcodes were unavailable for 148 children and were replaced with the child's school postcode. One child was missing both SDQ and EYFSP scores and six were missing EYFSP scores due to teachers exiting the on‐line screen before completion. The screen required a response to each individual item before teachers could progress to the next item, thus there were no further missing data.

Complete data sets on the language composites and nonverbal cognitive ability composite were available for 506/529 children participating in in‐depth assessment. Incomplete test data were largely attributed to child unable/unwilling to complete particular tasks (65.22% of incomplete test scores were from children with ‘no phrase speech’ who were unable to provide verbal responses to expressive language tasks), rendering the total score unreliable. In these cases (*n* = 22), the senior investigator (CN) used the completed test data to classify children as language impaired or not. Missing scores for these participants were not imputed, but the weights take account of these missing data. The weighted frequencies are therefore estimates for the whole mainstream school population in Year 1 (ages 5;1 – 6;10; excluding those with English as an additional language).

## Results

We present the estimated frequencies in the target‐screened population obtained after weighting for the design and nonparticipation effects (raw frequency counts are available in Table 1). The first analyses (Tables S1 and S2) identified children with existing medical diagnoses and/or intellectual disability. The estimated frequency was 307 children, of whom 151 also met our criteria for language disorder (prevalence: 2.34%, 95% CI: 1.40, 3.91). Table 1 reports prevalence estimates for language disorder of currently unknown origin, an estimated frequency of 488 (prevalence: 7.58% of population, 95% CI 5.33, 10.66). Of those children meeting criteria for language disorder, 309 had NVIQ scores within the average range (4.80%, 95% CI 3.06, 7.44), while 179 had low‐average scores (2.78%, 95% CI 1.57, 4.86). Note that our estimates do not include children already attending special schools for children with complex learning needs (less than 1% of the population at school entry) and thus slightly underestimate the total number of children with language disorder in the population.

**Table 1:** Prevalence estimates of language disorder of unknown origin using SCALES (DSM‐5) criteria, Tomblin et al. (1997) criteria and ICD‐10 criteria. Estimates for language impairment associated with known medical diagnosis/intellectual disability, and intellectual disability alone are provided for reference. NVIQ bands include ‘average’ (−1*SD* or greater), ‘low‐average’ (−2*SD* to −1*SD*) and ‘intellectual impairment (<−2*SD*)

	Raw number of children meeting criteria (denominator = 529)	Estimated numbers of children meeting criteria (denominator = 6,442)	Population prevalence in SCALES sample (% of population) (95% CIs)	Functional impact: % achieve ‘good level of development’ on EYFSP^g^	Functional impact: % abnormal behaviour on SDQ
Language disorder of unknown origin^a^ (total)	91	488	7.58 (5.33, 10.66)	11.8 (3.71, 31.71)	9.68 (5.43, 16.66)
*‘average’ NVIQ*	*54*	*309*	*4.80 (3.06, 7.44)*	9.00 (1.92, 33.37)	9.85 (4.66, 19.62)
*‘low‐average’ NVIQ*	*37*	*179*	*2.78 (1.57, 4.86)*	16.62 (2.96, 56.57)	9.38 (3.76, 21.54)
‘Specific Language Impairment’ Tomblin et al. (1997) criteria^b^	78	499	7.74 (5.38, 11.02)	27.60 (13.19, 48.88)	9.10 (4.96, 16.13)
‘Developmental Language Disorder’ ICD10 criteria^c^	14	69.2	1.07 (0.41, 2.82)	0	11.79 (2.57, 40.42)
Language impairment and known medical diagnosis/intellectual disability^d^	45	151	2.34 (1.40, 3.91)	14.73 (2.18, 57.24)	51.36 (27.55, 74.57)
Intellectual disability (NVIQ scores <−2*SD*)^e^	30	119	1.84 (0.97, 3.46)	18.9 (2.86, 64.81)	38.97 (13.49, 72.34)
Total language disorder^f^	136	639	9.92 (7.38, 13.20)	12.49 (4.70, 29.23)	19.55 (11.57, 31.08)

a DSM‐5 criteria: Language scores −1.5*SD* or more below normative mean on 2/5 language composite scores. NVIQ > 70, breakdown of average (above 85) and low‐average (between 70 and 85) in italic font. No known medical diagnosis.

b Tomblin et al. (1997): Language scores −1.25*SD* or more below normative mean on 2/5 language composite scores. NVIQ > 85. No known medical diagnosis. Note: inclusion of children with NVIQ > 70 increases prevalence estimate to 11.11%.

c ICD10 criteria: Language scores −2*SD* or more below normative mean on 2/5 language composite scores; NVIQ > 85, and no known medical diagnosis. Note this creates a significant (1*SD*) discrepancy between verbal and nonverbal ability.

d Breakdown of diagnoses given in Table S2.

e Includes both children meeting criteria for language impairment and those that did not.

f Total language disorder combines prevalence estimates from the SCALES DSM‐5 criteria and those with language impairments associated with a known medical diagnosis and/or intellectual disability. It does not include children with English as an additional language or children who started school in a specialist provision for children with severe and complex learning disabilities. This figure thus represents the minimum overall need for language‐based clinical/educational support.

g EYFSP: total achieving a ‘good level of development’ (GLD) in typically developing population is 69.59%.

For comparison, Table 1 also reports prevalence estimates using Tomblin et al. (1997) criteria (7.74%; the same criteria including children with low‐average NVIQ increases this estimate to 11.11%), and children meeting ICD‐10 discrepancy criteria (1.07%). For both SCALES criteria and Tomblin criteria, relaxing the NVIQ cut‐off score increases the prevalence estimate by approximately 50%. Most strikingly, very few children meet strict ICD‐10 criteria, largely because so many children obtain intermediate discrepancy scores.

Tables 1 and 2 also document the functional impairment associated with language disorder; 88% of children meeting SCALES criteria for language disorder failed to achieve a ‘good level of development’ on the EYFSP, compared with 30% of typically developing peers, *F*(1, 466) = 32.21, *p* < .001. Children with language disorder also displayed increased levels of social, emotional and behaviour difficulties, *F*(1, 466) = 7.78, *p* = .05. Table S2 compares these children to those for whom language impairment occurs in the context of a known diagnosis and/or intellectual disability. The latter group has significantly more severe language deficits and almost 50% have reported clinically significant social, emotional and behavioural deficits, yet the two groups did not differ significantly in terms of academic attainment, at least during the first year of school.

**Table 2:** Participant characteristics of those with language disorder versus typical language development. Estimated frequencies and means reported, with 95% CIs in parentheses. For categorical variables (indicated by %) the *F*‐statistic is a design based corrected *χ* ^2^ value

	TD (no known diagnosis)	Language Disorder (no known diagnosis)	*F* (1,466)	*p*
*N* raw (estimated)	376 (5,647)	91 (488)		
Gender ratio (M:F)	0.98:1	1.22:1		
% Male	47.88 (41.35, 54.48)	54.26 (36.73, 70.79)	0.44	.51
Age (months)	71.72 (71.09, 72.37)	71.52 (71.19, 72.85)	0.08	.78
IDACI rank	23,896 (22,939, 24,852)	16,243 (13,306, 19,179)	23.72	<.001
NVIQ composite (*z*‐score)	.14 (.02, .26)	−0.77 (−.98, −.56)	53.03	<.001
CCC‐S total (raw score)	7.04 (6.28, 7.80)	17.83 (14.78, 20.88)	45.48	<.001
SDQ‐Total Difficulties (raw score)	5.01 (4.39, 5.64)	7.28 (5.82, 8.75)	7.88	.005
% Social, emotional behavioural problems (SDQ ‘abnormal behaviour’)	5.24 (3.20, 8.47)	9.68 (5.43, 16.66)	2.67	.10
EYFSP total (raw score)	37.58 (36.63, 38.51)	28.79 (26.54, 31.04)	49.95	<.001
% achieving ‘good level of development’ (EYFSP)	69.59 (63.54, 75.03)	11.80 (3.71, 31.71)	32.21	<.001
% extra school support	6.82 (4.52, 10.18)	39.70 (24.53, 57.14)	36.12	<.001
% Statement of special educational need	0.12 (0.04, 0.41)	3.46 (1.46, 7.96)	46.39	<.001
% referral speech‐language therapy	10.48 (7.36–14.72)	39.03 (23.98, 56.51)	20.47	<.001
% consonants correct (speech)	98.96 (98.59, 99.33)	95.10 (93.16, 97.04)	14.77	<.001

IDACI, Income Deprivation Affecting Children Index; CCC‐S, Children's Communication Checklist‐Short; SDQ, Strengths and Difficulties Questionnaire; EYFSP, Early Years Foundation Stage Profile.

**Figure 2:**
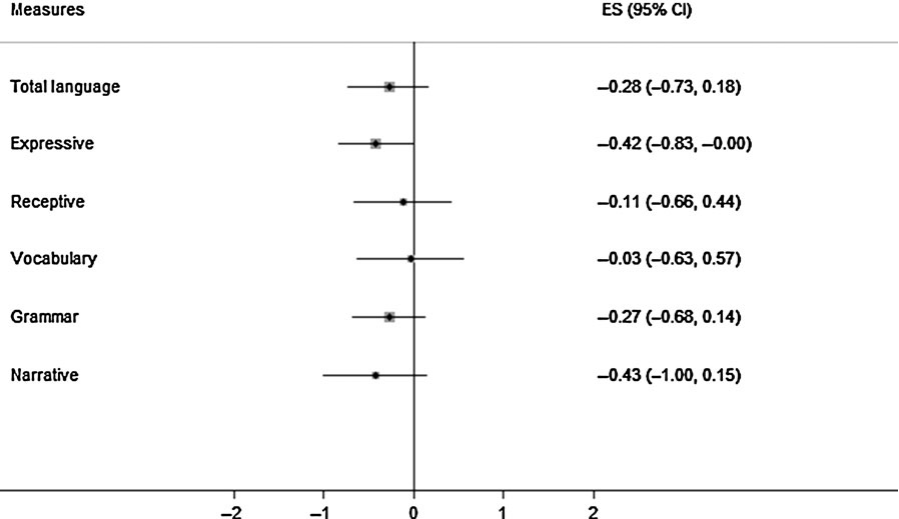
Standard *z*‐score differences (95% CI) between children with language disorder and low‐average NVIQ and those with average NVIQ on language composites. Error bars are 95% confidence intervals. Bars that cross the zero midline indicate no significant group difference. Boxes to the left of zero indicate poorer performance in the low‐average group

Figure 2 depicts the language profiles of children with language disorder (of currently unknown origin) by NVIQ band. In general, there were no differences in overall severity of language impairment. Those with lower NVIQ did have significantly more severe expressive language deficits, *F*(1, 90) = 4.01, *p *=* *.05, a composite score which is comprised of expressive vocabulary, sentence recall and narrative recall. Both sentence recall and narrative tasks tap memory skills as well as vocabulary and grammar and may therefore be particularly sensitive to broader cognitive deficits. There were, however, no differences between those with average and low‐average NVIQ with regard to any other language composite, age, socioeconomic status, symptom severity on the SDQ‐Total Difficulties scale or EYFSP total raw scores (Table 3). Thus, for children with language disorder, low‐average NVIQ was not associated with a more social disadvantage, a more severe language impairment, more severe social, emotional and behavioural problems or poorer academic attainment.

**Table 3:** Comparison of children with language disorder by NVIQ band. Weighted frequencies and means reported, with 95% CIs in parentheses. For categorical variables (indicated by %) the *F*‐statistic is a design based corrected *χ* ^2^ value

	‘Average’ NVIQ (NVIQ > −1*SD*)	‘Low‐average’ NVIQ (NVIQ between −2*SD* and −1*SD*)	*F* (1,90)	*p*
*N* raw (*N* weighted)	54 (309)	37 (179)		
Male:Female	1.56:1	0.81:1		
% Male	60.87 (38.22, 79.64)	44.71 (20.98, 71.13)	0.82	.37
Age (months)	72.26 (70.60, 73.92)	70.24 (68.68, 71.80)	3.10	.08
IDACI rank	15,317 (12,255, 18,380)	17,841 (11,833, 23,850)	0.55	.46
Total language composite (*z*‐score)	−1.60 (−1.77, −1.43)	−1.88 (−2.30, −1.46)	1.48	.23
CCC‐S total (raw score)	17.01 (13.12, 20.90)	19.25 (14.14, 24.35)	0.48	.49
SDQ‐Total difficulties (raw score)	7.24 (5.39, 9.09)	7.36 (4.87, 9.84)	0.01	.94
% Social, emotional behavioural problems (SDQ abnormal behaviour)	9.85 (4.61, 19.82)	9.38 (3.70, 21.81)	0.007	.93
EYFSP total (raw score)	29.53 (26.28, 32.78)	27.53 (25.16, 29.90)	0.97	.33
% achieving ‘good level of development’ (EYFSP)	9.00 (1.87, 33.95)	16.62 (2.88, 57.30)	0.31	.57
% School support	32.95 (16.43, 55.14)	51.35 (24.89, 77.07)	1.07	.30
% Statement of special educational need	3.07 (0.95, 9.45)	4.12 (1.09, 14.37)	0.11	.74
% referral speech‐language therapy	31.50 (15.36, 53.81)	52.05 (25.39, 77.59)	1.34	.25
% consonants correct (speech)	95.51 (92.89, 98.13)	94.37 (91.53, 97.22)	0.34	.56

IDACI, Income Deprivation Affecting Children Index; CCC‐S, Children's Communication Checklist‐Short; SDQ, Strengths and Difficulties Questionnaire; EYFSP, Early Years Foundation Stage Profile.

There were no significant sex differences in prevalence estimates for language disorder (1.22:1 males:females), despite significant sex differences at screening in which twice as many boys were identified as ‘high‐risk’. In contrast, the rate of language disorder associated with existing medical diagnosis and/or intellectual disability was much higher in males, 3.31:1 (Table S2). In general, for those with language disorder (in the absence of known diagnosis/intellectual impairment) there were no differences between males and females in severity of language composite scores, although there was a small, but significant difference in the grammar composite, in which girls obtained scores indicating more severe grammatical deficits (Figure S1).

Despite early and significant impacts on academic progress, fewer than half of those with language disorder were receiving extra help at school or had been referred to speech‐language therapy services (Table 2). The percentages of children receiving additional support varied widely within both NVIQ groups. Logistic regression identified female sex, severity of speech disorder and severity of language deficit as significantly associated with higher referral rates to speech‐language therapy (Table 4). Socioeconomic status, SDQ scores, EYFSP scores and NVIQ were not associated with referral. Children with language impairment in the context of known diagnoses and/or intellectual impairment were not any more likely to be referred to speech‐language therapy services, but a significantly greater percentage of children in this category (31%) were in receipt of a statement of special educational need relative to those with language disorder (3%). The types of support available vary considerably but most often involve time with a learning support assistant in the classroom.

**Table 4:** Logistic regression predicting referral to speech‐language therapy services from demographic and child variables. Female sex, severity of language disorder and increased number of speech sound errors significantly increase likelihood of referral to specialist speech‐language therapy services

	Odds ratio	*SE*	*p*	95% CI
Age (months)	1.04	0.04	.33	0.96, 1.14
Male sex	0.40	0.16	.02	0.19, 0.86
Income Deprivation Affecting Children Index (rank score)	1.00	0.00	.59	1.00, 1.00
Language composite (*z*‐score)	0.38	0.10	.00	0.23, 0.64
Speech (per cent consonant correct)	0.80	0.03	.00	0.74, 0.87
NVIQ composite (*z*‐score)	1.10	0.22	.65	0.74, 1.63
Strengths & Difficulties: total difficulties (raw score)	1.02	0.03	.52	0.96, 1.08
Early Years Foundation Stage Profile (raw score)	0.99	0.03	.78	0.94, 1.05
Constant	1.37	5.73	.00	3,656.69, 5.10e+10

The overall model is significant, Wald *χ* ^2^ (8) = 59.04, *p* < .001, and explained a significant, though modest, amount of variance (McFadden's pseudo *R* square = .28).

## Discussion

We estimated the prevalence of language disorder using a population screening procedure followed by a comprehensive assessment. Our prevalence estimate of 7.58% suggests that approximately two children in every Year 1 classroom of 30 children will have a clinically significant language disorder of currently unknown cause that adversely impacts learning. We also estimated the prevalence of language impairment associated with existing medical diagnoses and/or intellectual disorder (2.34%), a group with more severe language deficits and a high proportion of children with clinically significant social, emotional and behavioural difficulties. Our estimates are based on a population of children in mainstream classrooms and do not include children in special schools for children with complex learning needs or children with English as an additional language. In addition, our cohort is taken from a relatively affluent county in Southeast England. For these reasons our prevalence estimates should be considered the minimum estimate of need and may be higher in other areas.

Our study focused on prevalence, or the percentage of the target population experiencing language disorder at a given time, and did not measure incidence, which is the number of new cases of language disorder per population at risk within a given time period. Incidence rates are more difficult to estimate, but are important in understanding time trends and possible causes. However, it is noteworthy that despite more than 20 years between studies, different assessment measures and school environments, our prevalence estimate is broadly similar to the most widely cited epidemiological study (Tomblin et al., 1997), which used methods and language constructs similar to our own and which has informed DSM‐5 criteria for language disorder. A key difference between these two studies is that SCALES criteria used a more severe cut‐off score for language severity (−1.5*SD*) and included children with a broader range of nonverbal IQ scores, whereas Tomblin et al. only included children with NVIQ scores above 87. Employing the same language cut‐off as Tomblin et al., 1997, but increasing the NVIQ range to include all children who did not meet criteria for intellectual disability dramatically increased the prevalence estimate to 11.11%. This figure stands in stark contrast to the ICD‐10 estimate, in which children's language is below both age and cognitive ability, and a significant discrepancy between verbal and nonverbal abilities is required. Only 1.07% of children met these strict criteria; such criteria appear to lack face validity as many children with significant need would be excluded from such a diagnosis.

This study is the first to measure explicitly, at a population level, the impact of relaxing NVIQ criteria in the DSM‐5 on both prevalence and clinical profile of language disorder. Children with ‘low‐average’ NVIQ scores did not generally experience more severe language deficits, educational difficulties, or social, emotional and behavioural problems. Thus, there is no a priori reason to exclude such children from specialist clinical or educational services (Dockrell et al., 2006). Previous studies have highlighted low‐average NVIQ as a marker of persistent language disorder (Stothard, Snowling, Bishop, Chipchase, & Kaplan, 1998), associated with more severe academic challenges over time. We acknowledge that the causes of language disorder associated with concomitant low‐average NVIQ may be distinct and may warrant different treatment approaches. Intervention studies that systematically test the influence of NVIQ on response to treatment are therefore needed to develop best practice guidelines.

This study is also the first to include a measure of functional impact, a nationally applied measure of academic attainment. There was a clear association between SCALES criteria for language disorder and academic disadvantage, with only 11% of affected children achieving curriculum targets in the first year of school (14% of those with language impairment in the context of a known diagnosis and/or intellectual impairment). Longitudinal investigations have reported increased risk for literacy disorders and continued scholastic underachievement, often associated with higher rates of emotional, social and behavioural deficit (Tomblin, Zhang, Buckwalter, & Catts, 2000). Thus, developing language skills that enable children to access the curriculum and support social, emotional and behavioural development is a key priority for clinical services.

We also acknowledge that we operationalized DSM‐5 criteria to require disorder in two of five language composite scores and used a more severe, but still arbitrary, cut‐off for language disorder at −1.5*SD* (or approximately the bottom 7th centile). This cut‐off score is consistent with identification of language disorder in other clinical conditions (Loucas et al., 2008) and in this sample is closely aligned with functional impairment in school attainment. In comparison, the more lenient cut‐off employed by Tomblin et al. (1997) identified a large number of false positives, and in the current sample 2/3 of children meeting these criteria exhibited associated functional impacts. While none of the children meeting ICD‐10 diagnostic criteria achieved early curriculum targets, the number of children who met criteria was too low and excluded too many children with language and learning needs to be clinically meaningful. Nevertheless, longitudinal follow‐up of the current cohort will be essential to establishing the diagnostic framework with the most appropriate criteria for identifying children with persistent language disorders and associated functional impacts.

The sex ratio (1.22:1, male:female) for language disorder is similar to previous epidemiological reports (Beitchman et al., 1986; Tomblin et al., 1997), although the sex ratio for language impairment associated with known medical diagnoses and/or intellectual disorder is much higher, due in part to the high percentage (33%) of children with autism diagnoses within this group. Despite similar rates and severity of language disorder between sexes, there were differences in identification and referral to clinical services. While more boys were identified at screening as being at risk, girls were more likely to be referred to clinical services. We did take a sex‐specific cut‐off on the CCC‐S, largely to account for the confounds between sex, age group and teacher ratings at screening. Had we not done this, our intensively assessed cohort would have included a large proportion of summer‐born boys, many of whom would likely have resolved early language delays. Nevertheless, our sample weighting procedures take account of this and thus the reported scores reflect gender distributions in the screened population. Therefore, the differences in assessed outcome (which did not use sex‐specific cut‐offs) or referral patterns are not due to sampling methods. The reasons for these discrepancies are uncertain, but suggest the need for a more systematic approach and increased training of health and education professionals regarding the symptom profile of language disorder in both sexes.

### Study limitations

Our study uniquely measured functional impact in a representative sample, reducing potential influence of referral bias (Berkson's bias) that is evident in clinically referred samples. Nevertheless, our study is limited by the exclusion of children with English as an additional language (10.7% of the screened population). There were 64 different languages represented in the cohort and it was not possible to obtain estimates of language ability in both English and the child's home language. Our population is also more affluent than the national average, although we sampled from across the social strata. Approximately 10% of school‐aged pupils in Surrey are privately educated and are not represented in our sample, largely because such schools are not obliged to report national curriculum assessments and thus we would be unable to map functional impact of language disorder for these children. It is unknown what proportion of those children in private schools experience language disorder. At the opposite end of the spectrum, we had fewer children from impoverished neighbourhoods than the national average. Our findings suggest a small, but significant association between lower socioeconomic status and language disorder, thus prevalence rates are likely to be higher in areas of the country with increased socioeconomic disadvantage. Although we had direct measures of language and NVIQ, our measures of social, emotional and behavioural problems and educational attainment relied exclusively on teacher report. Direct observation in combination with parental report of functional impact would provide a more holistic view of the child's strengths and clinical needs. We obtained information about referral to speech‐language therapy, but it was not possible to obtain accurate information about ongoing involvement of specialist clinicians. Finally, we were only able to directly assess language ability and functional impact at one point in time, while some authorities have advocated assessment at two points for reliable identification (Reilly, Tomblin, et al., 2014). We note that this recommendation refers primarily to preschool children and that stability of language disorder is greater after school entry (Tomblin et al., 2003).

## Conclusions

Current DSM‐5 criteria for language disorder does not require a minimum level of nonverbal cognitive ability and yields a prevalence estimate that is at least seven times higher than the ICD‐10 estimate, which requires both NVIQ within the normal range and significant discrepancy between verbal and nonverbal ability. Importantly, our diagnostic criteria identify children for whom the majority experience functional impact on learning in the first year of formal schooling. We found minimal differences in the language and clinical profiles of those with average versus low‐average NVIQ, supporting the decision to remove NVIQ, and particularly the discrepancy between verbal and nonverbal abilities, as exclusion criteria for developmental language disorder in DSM‐5. Notably, children who experience language impairment as part of a known medical condition and/or intellectual disorder tend to have more severe language disorders and more pervasive developmental concerns. Nevertheless, these children would also likely benefit from specialist clinical input in order to maximize communication and learning. It is now imperative that children with varying nonverbal cognitive profiles are included in intervention trials to provide much needed evidence concerning response to treatment at different levels of NVIQ. This study also emphasizes the need to raise awareness among education and health services regarding language disorder and its functional impact on children's daily lives.

**Key points**

- Language disorder is a common cause of referral to health services in childhood and significantly increases risk for long‐term learning, social, emotional and behavioural problems.
- NVIQ is frequently used as an exclusion criteria, preventing children from accessing specialist clinical services.
- We report the first population prevalence estimates and associated functional impacts of language disorder using DSM‐5 criteria.
- At school entry, 7.58% of children have clinically significant language disorder of unknown cause associated with increased rates of social, emotional and behavioural difficulties, and academic underachievement.
- The profile and severity of language disorder and associated functional impacts are similar in children with average and below average NVIQ scores.
- Access to specialist clinical and educational services (i.e. speech‐language therapy) should not depend on level of NVIQ.

## Supporting information


**Appendix S1.** Core test battery administered at Phase 2.
**Appendix S2.** STROBE Statement – Checklist of items that should be included in reports of cohort studies.
**Table S1.** Unweighted frequencies of children with known clinical diagnoses or intellectual impairment as reported by teachers in Phase 1 or Phase 2 or by Phase 2 in‐depth assessment.
**Table S2.** Characteristics of participants meeting criteria for language disorder with an existing medical diagnosis and/or intellectual impairment (left column) and those meeting criteria for language disorder of unknown origin (right column). For categorical variables (indicated by %) the F‐statistic is a design based corrected *χ*2 value.
**Figure S1.** Standard score difference between males and females with language disorder of unknown origin on nonverbal IQ and language composites (error bars are 95% confidence intervals). Bars that cross the zero midline indicate no group difference. Boxes to the left of zero indicate poorer performance by females.
