# Supplementary material for: The impact of nonverbal ability on prevalence and clinical presentation of language disorder: evidence from a population study
Source: J Child Psychol Psychiatry. 2016 May 16;57(11):1247–57. doi: 10.1111/jcpp.12573 (PMC5082564; doi:10.1111/jcpp.12573)

Supplemental Material.

Norbury et al. The impact of non-verbal ability on prevalence and clinical presentation of Language Disorder: evidence from a population study

**Appendix S1.** Core test battery administered at Phase 2.

**Non-verbal IQ**

*WISC-IV Block Design.* Each child was asked to copy patterns, presented to them in a stimulus booklet, using red and white blocks. Trials were scored as correct or incorrect, time taken to complete pattern recorded and items scored according to the manual (maximum score = 68). The average corrected stability coefficient is good (>0.80s).

*WISC-IV Matrix Reasoning.* The child was required to select a picture, from a choice of five that best completed the matrix. For each correct response the child was credited with one point (maximum score = 35). Matrix Reasoning has an excellent average test-retest reliability (r = .85) and internal consistency (r = .89).

**Core language battery**

Children completed six assessments of receptive and expressive skills across multiple domains of language (vocabulary, grammar and narrative; Tomblin, Records, Buckwalter, Zhang, Smith, & O'Brien, 1997).

1. *Receptive One word Picture Vocabulary Test (ROWPVT-4; Martin & Brownell, 2000*). The child heard a word and had to select the corresponding picture, from a choice of four (maximum score = 190). The manual reports excellent internal consistency for ages 5- to 8-years (Cronbach’s α =.95 – .97). Test-retest reliability coefficients are 0.97 (raw scores) and 0.91 (standard scores).
2. *Expressive One Word Picture Vocabulary Test (EOWPVT; Martin & Brownell, 2000).* The child was asked to name coloured drawing of objects, actions or concepts (maximum score= 190). As before, Internal consistency for ages 5- to 8-years is excellent (Cronbach’s α = .94 – .97), as is test-retest reliability: 0.98 (raw scores) and 0.97 (standard scores) (Martin & Brownell. 2011).
3. *Test of Reception of Grammar – Short Form (TROG-S).* Forty of the original 80 items were included in the short form (Blocks: F, G, H, K, L, N, P, O, S, T). Children heard a sentence such as “the ball that is red is on the pencil” and were asked to select the corresponding picture from a choice of four. Testing was discontinued after six consecutive incorrect responses. Total items correct, rather than total correct blocks was the final score (maximum score = 40). Pilot testing demonstrated excellent agreement between short and long forms of *r*(17) = 0.88.
4. *School-aged sentence imitation test- E32 (Marinis et al. 2011).* Thirty-two sentences of increasing length and grammatical complexity were pre-recorded by a female, native speaker of English and played over headphones to the child one at a time. Children were asked to repeat each sentence verbatim and their responses were digitally recorded. Items were scored for overall accuracy (maximum score = 32).
5. *Assessment of Comprehension and Expression 6-11 (ACE 6-11; Adams et al., 2001).*  The *Narrative Recall* required the child to listen to a story about a monkey in a forest. The story was pre-recorded by a female, native speaker of English and played over headphones while the accompanying pictures were displayed on a laptop computer. This controlled for potential administrator differences in speed of presentation and prosodic differences. After listening to the story, the child was asked to retell it while viewing the pictures. The child’s story was audio recorded and scored according to the number of key information units contained in the story (range 0 to 35). According to the manual, the internal consistency is adequate (Cronbach’s α = .73) for children aged 6- to 11-years.
6. *Narrative Comprehension*. Following the Narrative Recall task, the child was asked to answer 12 bespoke comprehension questions (6 literal and 6 inference questions). Reponses were scored 0 for an incorrect answer, 1 point for a partially correct response and 2 points for a correct response (maximum score = 24), using a written guide developed for this study. All scoring was done by consensus to ensure consistency of scoring.

*Vocabulary Composite* combines a and b

*Grammar Composite* combines c and d

*Narrative composite* combines e and f

*Receptive Language Composite* combines a, c, and e

*Expressive Language Composite* combines b, d, and f

*Total Language Composite.* Sum of all test scores, independently standardised using the LMS procedure.

**Speech**

*Diagnostic Evaluation of Articulation and Phonology* (*DEAP*; Dodd, Hua, Crosbie, Holm & Ozanne, 2002). The child was asked to name 30 coloured drawings that tapped a range of English phonemes. If the child was unable to produce the word spontaneously, they were asked to imitate the word which was said by the researcher. The task was audio-recorded and errors scored (maximum errors = 62). Percent consonants correct was calculated as: (total accurate consonant productions / 62)*100.

**Appendix S2.** STROBE Statement—Checklist of items that should be included in reports of cohort studies.

|  | Item No | Recommendation |
| --- | --- | --- |
| **Title and abstract** | 1 | (*a*) Indicate the study’s design with a commonly used term in the title or the abstract X |
|  |  | (*b*) Provide in the abstract an informative and balanced summary of what was done and what was found X |
| Introduction | | |
| Background/rationale | 2 | Explain the scientific background and rationale for the investigation being reported: pages 6-7 |
| Objectives | 3 | State specific objectives, including any prespecified hypotheses: abstract and page 8 |
| Methods | | |
| Study design | 4 | Present key elements of study design early in the paper: abstract and pages 8-11 |
| Setting | 5 | Describe the setting, locations, and relevant dates, including periods of recruitment, exposure, follow-up, and data collection: pages 8-11 and figure 1 |
| Participants | 6 | (*a*) Give the eligibility criteria, and the sources and methods of selection of participants. Describe methods of follow-up: page 8, figure 1 |
|  |  | (*b*) For matched studies, give matching criteria and number of exposed and unexposed N/A |
| Variables | 7 | Clearly define all outcomes, exposures, predictors, potential confounders, and effect modifiers. Give diagnostic criteria, if applicable: pages 9-10 |
| Data sources/ measurement | 8* | For each variable of interest, give sources of data and details of methods of assessment (measurement). Describe comparability of assessment methods if there is more than one group. Pages 9-10 |
| Bias | 9 | Describe any efforts to address potential sources of bias: page 8 |
| Study size | 10 | Explain how the study size was arrived at: page 8-9 |
| Quantitative variables | 11 | Explain how quantitative variables were handled in the analyses. If applicable, describe which groupings were chosen and why: page 11 |
| Statistical methods | 12 | (*a*) Describe all statistical methods, including those used to control for confounding |
|  |  | (*b*) Describe any methods used to examine subgroups and interactions |
|  |  | (*c*) Explain how missing data were addressed : page 11 |
|  |  | (*d*) If applicable, explain how loss to follow-up was addressed: N/A, but percentages of children coming from screen phase to phase 2 is on page 10 |
|  |  | (*e*) Describe any sensitivity analyses: N/A |
| Results | | |
| Participants | 13* | (a) Report numbers of individuals at each stage of study—eg numbers potentially eligible, examined for eligibility, confirmed eligible, included in the study, completing follow-up, and analysed: pages 9&10 |
|  |  | (b) Give reasons for non-participation at each stage: pages 9-11 |
|  |  | (c) Consider use of a flow diagram: Figure 1 |
| Descriptive data | 14* | (a) Give characteristics of study participants (eg demographic, clinical, social) and information on exposures and potential confounders:Tables 2 & 3 |
|  |  | (b) Indicate number of participants with missing data for each variable of interest pg 11. |
|  |  | (c) Summarise follow-up time (eg, average and total amount): page 9 |
| Outcome data | 15* | Report numbers of outcome events or summary measures over time N/A |
| Main results | 16 | (*a*) Give unadjusted estimates and, if applicable, confounder-adjusted estimates and their precision (eg, 95% confidence interval). Make clear which confounders were adjusted for and why they were included : page 12, Table 1-3 Figure 2 |
|  |  | (*b*) Report category boundaries when continuous variables were categorized, Table 1 |
|  |  | (*c*) If relevant, consider translating estimates of relative risk into absolute risk for a meaningful time period N/A |
| Other analyses | 17 | Report other analyses done—eg analyses of subgroups and interactions, and sensitivity analyses. Table 3 |
| Discussion | | |
| Key results | 18 | Summarise key results with reference to study objectives. Pages 13-14 |
| Limitations | 19 | Discuss limitations of the study, taking into account sources of potential bias or imprecision. Discuss both direction and magnitude of any potential bias. Page 14 |
| Interpretation | 20 | Give a cautious overall interpretation of results considering objectives, limitations, multiplicity of analyses, results from similar studies, and other relevant evidence. Page 15 |
| Generalisability | 21 | Discuss the generalisability (external validity) of the study results. Page 15 |
| Other information | | |
| Funding | 22 | Give the source of funding and the role of the funders for the present study and, if applicable, for the original study on which the present article is based. Page 11-12 |

*Give information separately for exposed and unexposed groups.

**Note:** An Explanation and Elaboration article discusses each checklist item and gives methodological background and published examples of transparent reporting. The STROBE checklist is best used in conjunction with this article (freely available on the Web sites of PLoS Medicine at http://www.plosmedicine.org/, Annals of Internal Medicine at http://www.annals.org/, and Epidemiology at http://www.epidem.com/). Information on the STROBE Initiative is available at http://www.strobe-statement.org.

**Table S1.** Unweighted frequencies of children with known clinical diagnoses or intellectual impairment as reported by teachers in Phase 1 or Phase 2 or by Phase 2 in-depth assessment.

| Primary diagnosis | Unweighted frequency | N males |
| --- | --- | --- |
| Hearing impairment | 3 | 2 |
| Visual Impairment | 2 | 1 |
| ASD | 20 | 18 |
| Epilepsy | 6 | 3 |
| Head injury/Neurological impairment | 2 | 1 |
| Cerebral Palsy | 1 | 1 |
| Down syndrome | 2 | 1 |
| Noonan syndrome | 1 | 1 |
| Neurofibromatosis | 1 | 1 |
| Intellectual Disability, no diagnosis (<=-2SD on tests) | 22 | 14 |
| Total | 60 | 43 |

**Table S2.** Characteristics of participants meeting criteria for language impairment with an existing medical diagnosis and/or intellectual impairment (left column) and those meeting criteria for language impairment of unknown origin (right column). For categorical variables (indicated by %) the *F*-statistic is a design based corrected χ^2^ value.

|  | Language Impairment+ known diagnosis/ intellectual impairment | Language impairment unknown origin | F  (1, 35) | p-value |
| --- | --- | --- | --- | --- |
| N raw (estimated) | 45 (151) | 91 (488) |  |  |
| Prevalence | 2.34% | 7.57% |  |  |
| Gender ratio (M:F) | 3.31:1 | 1.22:1 |  |  |
| % Male | 76.84 | 54.95 | 3.27 | .07 |
|  | (58.71, 88.56) | (37.33, 71.41) |  |  |
| Age (months) | 71.52 | 73.08 | 2.68 | .10 |
|  | (70.18, 74.40) | (71.76, 74.39) |  |  |
| IDACI rank | 18923 | 16243 | 0.94 | .33 |
|  | (14330, 23516) | (13280, 19206) |  |  |
| Total language composite  (z-score)* | -2.16  (-2.49, -1.83) | -1.70  (-2.16, -1.51) | 5.59 | .02 |
|  |  |  |  |  |
| Non-verbal IQ composite (z-score)* | -1.82  (-2.23, -1.41) | -0.77  (-0.98, -0.55) | 20.34 | <.001 |
|  |  |  |  |  |
| CCC-S total (raw score)* | 25.24 | 17.83 | 6.46 | .012 |
|  | (20.37, 30.10) | (14.76, 20.91) |  |  |
| SDQ total difficulties (raw score)* | 14.21  (9.41, 19.01) | 7.29  (5.81-8.76) | 7.44 | .007 |
|  |  |  |  |  |
| % Social, emotional behavioural problems* (SDQ ‘abnormal’) | 51.36  (27.55, 74.57) | 9.68  (5.43, 16.65) | 17.08 | <.001 |
|  |  |  |  |  |
| EYFSP total (raw score) | 25.79 | 28.79 | 0.84 | .36 |
|  | (19.70, 31.88) | (26.52, 31.07) |  |  |
| % achieving ‘good level of development’ (EYFSP) | 14.73  (2.18, 57.24) | 11.80  (3.67, 31.95) | 0.04 | .83 |
|  |  |  |  |  |
| % School support | 61.20 | 39.70 | 2.32 | .13 |
|  | (38.93, 79.60) | (24.42, 57.29) |  |  |
| % Statement of special educational need* | 30.81  (15.92, 51.14) | 3.46  (1.45, 8.02) | 22.91 | <.001 |
|  |  |  |  |  |
| % referral speech-language therapy | 66.18  (35.89, 87.24) | 39.03  (23.86, 56.67) | 2.47 | .12 |
| % consonants correct (speech) | 93.79  (90.52, 97.06) | 95.10  (93.14, 97.06) | 0.46 | .50 |

IDACI: Income Deprivation Affecting Children Index; CCC-S: Children’s Communication Checklist-Short; SDQ: Strengths and Difficulties Questionnaire; EYFSP: Early Years Foundation Stage Profile. Note: * denotes difference between means or proportions at p < .05.

**Figure S1.** Standard score difference between males and females with Language Disorder of unknown origin on non-verbal IQ and language composites (error bars are 95% confidence intervals). Bars that cross the zero mid-line indicate no group difference. Boxes to the left of zero indicate poorer performance by females.


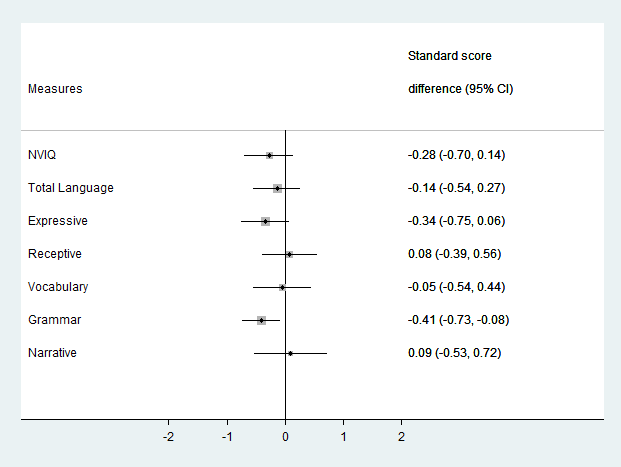

Supplement: Supplementary file 1 — Appendix S1. Core test battery administered at Phase 2. Appendix S2. STROBE Statement – Checklist of items that should be included in reports of cohort studies. Table S1. Unweighted frequencies of children with known clinical diagnoses or intellectual impairment as reported by teachers in Phase 1 or Phase 2 or by Phase 2 in‐depth assessment. Table S2. Characteristics of participants meeting criteria for language disorder with an existing medical diagnosis and/or intellectual impairment (left column) and those meeting criteria for language disorder of unknown origin (right column). For categorical variables (indicated by %) the F‐statistic is a design based corrected χ2 value. Figure S1. Standard score difference between males and females with language disorder of unknown origin on nonverbal IQ and language composites (error bars are 95% confidence intervals). Bars that cross the zero midline indicate no group difference. Boxes to the left of zero indicate poorer performance by females. [file JCPP-57-1247-s001.docx]
